# Outcomes of preexisting diabetes mellitus in breast, colorectal, and prostate cancer

**DOI:** 10.1007/s11764-017-0631-2

**Published:** 2017-07-22

**Authors:** Robert I. Griffiths, José M. Valderas, Emily C. McFadden, Clare R. Bankhead, Bernadette A. Lavery, Nada F. Khan, Richard J. Stevens, Nancy L. Keating

**Affiliations:** 1 Nuffield Department of Primary Care Health Sciences, University of Oxford, Radcliffe Primary Care Building, Radcliffe Observatory Quarter, Woodstock Road, Oxford, OX2 6GG UK; 2 Division of General Internal Medicine, Johns Hopkins University School of Medicine, Baltimore, 21205 USA; 3 Health Services & Policy Research Group and Exeter Collaboration for Primary Care (APEx), University of Exeter, Exeter, EX4 4SB UK; 4 Oxford University Hospitals NHS Trust, Oxford, OX3 9DU UK; 5 Thames Valley Strategic Clinical Network, NHS England, Leeds, UK; 6 Royal Liverpool University Hospital, L7 8XP, Liverpool, UK; 7 Department of Health Care Policy, Harvard Medical School, Boston, 02115 USA; 8 Division of General Internal Medicine and Primary Care, Brigham and Women’s Hospital, Boston, 02115 USA

**Keywords:** Diabetes mellitus, Breast neoplasms, Colorectal neoplasms, Prostatic neoplasms, Outcome assessment (health care), Diabetes complications, Mortality, Epidemiology

## Abstract

**Purpose:**

Preexisting diabetes is associated with increased morbidity and mortality in cancer. We examined the impact of incident cancer on the long-term outcomes of diabetes.

**Methods:**

Using the United Kingdom Clinical Practice Research Datalink, we identified three cohorts of diabetes patients subsequently diagnosed with breast, colorectal, or prostate cancer, each matched to diabetic noncancer controls. Patients were required to have survived at least 1 year after cancer diagnosis (cases) or a matched index date (controls), and were followed up to 10 years for incident microvascular and macrovascular complications and mortality. Multivariate competing risks regression analyses were used to compare outcomes between cancer patients and controls.

**Results:**

Overall, there were 3382 cancer patients and 11,135 controls with 59,431 person-years of follow-up. In adjusted analyses, there were no statistically significant (*p* ≤ 0.05) differences in diabetes complication rates between cancer patients and their controls in any of the three cancer cohorts. Combined, cancer patients were less likely (adjusted hazard ratio [HR] 0.88; 95% CI = 0.79–0.98) to develop retinopathy. Cancer patients were more likely to die of any cause (including cancer), but prostate cancer patients were less likely to die of causes associated with diabetes (HR 0.61; 95% CI = 0.43–0.88).

**Conclusions and implications:**

There is no evidence that incident cancer had an adverse impact on the long-term outcomes of preexisting diabetes.

**Implications for Cancer Survivors:**

These findings are important for cancer survivors with preexisting diabetes because they suggest that substantial improvements in the relative survival of several of the most common types of cancer are not undermined by excess diabetes morbidity and mortality.

## Introduction

Evidence from epidemiologic studies, as summarized in a number of recent reviews [1–10], indicates that preexisting diabetes is associated with worse short- and long-term outcomes of cancer, both overall [9, 10] and in specific types of cancer including breast [3, 4, 10], colorectal [2–7, 10], and prostate [8]. There are several reasons why the converse also may be true—that incident cancer adversely impacts the outcomes of preexisting diabetes. Potential mechanisms include adverse effects of cancer treatments on glycemic control [11, 12], impact of cancer on patient self-management of diabetes [13], and changes in the quality of diabetes primary care services during cancer treatment and follow-up [14–19]. However, there is considerably less information on how cancer impacts the long-term outcomes of preexisting diabetes [12, 20].

This gap in our understanding of how cancer impacts preexisting diabetes is important from several perspectives. For instance, as early detection and advances in cancer therapy and supportive care have substantially improved the relative survival of many of the most common types of cancer [21], overall morbidity and mortality in cancer depend increasingly on the quality and outcomes of primary care for other underlying conditions [22]. In response, cancer organizations, such as Cancer Research UK and Macmillan Cancer Support in the United Kingdom (UK), have expressed concern that overlooking other medical conditions during cancer treatment and follow-up could result in excess morbidity and mortality, thereby undermining gains associated with early detection and treatment of cancer [23, 24].

The objective of this study was to examine the effect of cancer on the long-term outcomes of preexisting diabetes.

## Methods

### Study design and data source

We conducted a historical cohort study using the UK Clinical Practice Research Datalink (CPRD) linked to the Office for National Statistics (ONS) mortality data. The CPRD contains anonymized information from general practitioner (GP) practices on demographics, symptoms, tests, diagnoses, therapies, health-related behaviors, and referrals to secondary care for over 11.3 million patients from 674 practices in the UK [25]. There are 4.4 million active (alive, currently registered) patients in the database, which is approximately 6.9% of the UK population. These patients are broadly representative of the UK general population in terms of age, sex, and ethnicity [25]. This database (including its predecessor, the General Practice Research Datalink) has been used extensively for health services and epidemiologic research in cancer and diabetes [26–30].

### Patient selection

Using CPRD, we identified three cohorts of cancer patients with preexisting diabetes, each matched to diabetic noncancer controls. Cancer patients (cases) were included if they met all of the following criteria: (a) diagnosed with breast, colorectal, or prostate cancer on or after January 1st, 2000; (b) diagnosed with type I or type II diabetes at least 2 years before their date of cancer diagnosis (index date); (c) had no other cancer diagnosis, except nonmelanoma skin cancer, before their index date; (d) were age ≥ 50 years at their index date; (e) had at least 2 years of eligible CPRD data before their index date; (f) had an index date before the end of the eligible CPRD data; and (g) survived and were otherwise eligible for follow-up 1 year after cancer diagnosis. Patients were required to have been age ≥ 50 years at their index date because diabetes in older adults is linked to higher mortality [31] and because older adults with diabetes are at substantial risk for both acute and chronic microvascular and cardiovascular complications [32]. Men with breast cancer were excluded. Read codes from the Department of Health, Data and Business (QOF) Rules, Cancer and Diabetes Indicator Sets, version 25.0 [33], were used to identify diabetes and cancer in CPRD.

#### Control selection: matching

Each cancer patient (case) was matched to up to four noncancer patients (controls) with preexisting diabetes on GP practice number, sex (colorectal only), and age (±1 year) at cancer diagnosis. Matched controls were also required to have met inclusion criteria d–g above. In addition to the three cohorts of breast, colorectal, and prostate cancer patients plus noncancer controls, a fourth cohort was constructed by combining the three individual cohorts.

Patients were followed up to 10 years after their index date for new microvascular and macrovascular complications (as described below). They were followed from 1 year after their index date up to 10 years for all-cause and diabetes mortality.

### Variables

#### Diabetes complications

Complications of diabetes consisted of incident microvascular and macrovascular conditions [34] first identified in patients’ electronic health records up to 10 years after their index date. Microvascular conditions consisted of retinopathy, neuropathy, nephropathy, chronic kidney disease (stage 4 or 5), and the composite outcome of any of the four above. Macrovascular complications consisted of peripheral arterial disease, acute myocardial infarction or coronary syndrome, cerebrovascular accident, lower limb amputation, and the composite outcome of any of the four above. Incident complications were identified using published lists of Read codes [35] present in patients’ records up to 10 years after their index date. Those patients identified with a specific complication prior to their index date were excluded from the population at risk for that complication during follow-up.

#### Mortality

Variables were constructed for all-cause and diabetes mortality, which was defined as the presence of an International Classification of Diseases, 10th Revision, Clinical Modification (ICD-10-CM) code in the ONS data indicating that the underlying cause of death was due to diabetes (ICD-10-CM E10–14), hyperglycemia (R73), hypoglycemia (E16.1, E16.2), myocardial infarction (I21–I22), ischemic heart disease (I20, I24, I25), stroke/sequelae (I60–I64, I69.0–I69.4), heart failure (I50), sudden death due to cardiac arrest (I46), peripheral vascular disease (I70–I74), or kidney disease (N00–N28) [36, 37].

#### Covariates

Baseline characteristics consisted of age at index date, sex, calendar year of index date, most recent (before the index date) smoking status, most recent drinking status, and index of multiple deprivation quintile, from least deprived (=1) to most deprived (=5); body mass index (kg/m^2^); and Charlson comorbidity index [38, 39]. Baseline clinical and laboratory values consisted of blood pressure (mm Hg), total cholesterol (mmol/L), and glycosylated hemoglobin (HbA1c: mmol/mol), identified with the use of the most recent value within 1 year before the index date. Categorical variables for laboratory values were constructed by using cutoffs that corresponded to the thresholds for meeting the laboratory-based performance indicators in the QOF Rules, Diabetes Indicator Set, version 25.0 [33]: blood pressure ≤140/80 mm Hg, total cholesterol ≤5 mmol/L (193 mg/dL), and HbA1c ≤59 (7.5%), 59–≤64 (8.0%), 64–≤75 (9.0%), and >75 mmol/mol. Baseline antidiabetic agents were identified with the use of the British National Formulary codes in the CPRD therapy file [40] within 1 year before the index date.

### Statistical methods

Since there was a reasonable chance overall mortality would be higher in cancer patients than controls, we used competing risks regression according to the approach proposed by Fine and Gray [41] to estimate the cumulative incidence function and unadjusted and adjusted hazard ratios (HR) for each microvascular and macrovascular complication, comparing cancer patients to noncancer controls. Patients were censored at 10 years after their index date or at the end of their eligibility for follow-up in the data, whichever came first. The competing risk was death prior to the date of censoring. Adjusted analyses included baseline demographic and clinical covariates as described above. Patients with a specific diabetes complication prior to their index date were excluded from that particular analysis.

Competing risks regression [41] also was used to estimate unadjusted and adjusted HRs for all-cause and diabetes mortality. Only patients eligible for linkage to the ONS data were included in the mortality analyses. Also, since study inclusion criteria required patients to have survived at least 1 year after their index date, survival analyses included only years 2–10 of follow-up.

## Results

Overall, there were 14,517 patients in the combined cohort: 3382 (23.3%) cancer patients and 11,135 (76.7%) controls (Table 1). There were no statistically significant differences in age, sex, and year of diagnosis between cancer patients and controls, because age and sex were used as matching criteria in constructing the cohorts, and controls received the same index date as their corresponding cases. There were small but statistically significant (*p* ≤ 0.05) differences between cancer patients and controls in the distributions of smoking status, BMI, and Charlson comorbidity index (Table 1). There were no differences between cancer patients and controls in mean baseline blood pressure or HbA1c. Cancer patients had statistically significantly lower mean baseline total cholesterol than controls (Table 1). However, the absolute difference was only 0.1 mmol/L (3.9 mg/dL).

**Table 1:** Patient characteristics—combined cohorts

	Combined cohorts						*p* value
	Cancer (*n* = 3382)		Control (*n* = 11,135)		All (*n* = 14,517)		
	*n*	%	*n*	%	*n*	%	
Age							
50–<60	248	7.3	800	7.2	1048	7.2	0.740
60–<70	946	28.0	3098	27.8	4044	27.9	
70–<80	1498	44.3	5041	45.3	6539	45.0	
≥80	690	20.4	2196	19.7	2886	19.9	
Sex							
Male	1974	58.4	6551	58.8	8525	58.7	0.630
Female	1408	41.6	4584	41.2	5992	41.3	
Year of diagnosis							
2000–2004	546	16.1	1649	14.8	2195	15.1	0.087
2005–2009	1500	44.4	4902	44.0	6402	44.1	
≥2010	1336	39.5	4584	41.2	5920	40.8	
Smoking status							
Nonsmoker	991	29.3	3364	30.2	4355	30	<0.001
Ex-smoker	1716	50.7	5633	50.6	7349	50.6	
Current smoker	324	9.6	1240	11.1	1564	10.8	
Not reported	351	10.4	898	8.1	1249	8.6	
Body mass index							
<25	579	17.1	1932	17.4	2511	17.3	<0.001
25–<30	1272	37.6	4183	37.6	5455	37.6	
≥30	1344	39.7	4656	41.8	6000	41.3	
Not reported	187	5.5	364	3.3	551	3.8	
Charlson comorbidity index							
1–2	1878	55.5	5898	53.0	7776	53.6	0.024
3–4	1044	30.9	3578	32.1	4622	31.8	
>4	460	13.6	1659	14.9	2119	14.6	
Type of cancer (or control)							
Breast	1036	30.6	3194	28.7	4230	29.1	<0.001
Colorectal	1069	31.6	4047	36.3	5116	35.2	
Prostate	1277	37.8	3894	35.0	5171	35.6	
Type of diabetes							
Type I	141	4.2	554	5.0	695	4.8	0.054
Type II	3241	95.8	10,581	95.0	13,822	95.2	
Any microvascular complication							
No	2412	71.3	7750	69.6	10,162	70.0	0.056
Yes	970	28.7	3385	30.4	4355	30.0	
Any macrovascular complication							
No	2681	79.3	8684	78.0	11,365	78.3	0.113
Yes	701	20.7	2451	22.0	3152	21.7	
Any antidiabetic agent							
No	769	22.7	2363	21.2	3132	21.6	0.060
Yes	2613	77.3	8772	78.8	11,385	78.4	
Blood pressure ≤140/80 mm Hg							
Yes	2026	59.9	6770	60.8	8796	60.6	<0.001
No	1250	37.0	4252	38.2	5502	37.9	
Not reported	106	3.1	113	1.0	219	1.5	
Systolic mean (SD)	3276	136 (15)	11,022	136 (16)	14,298	136 (16)	0.205
Diastolic mean (SD)	3276	74 (9)	11,022	74 (9)	14,298	74 (9)	0.090
Total cholesterol ≤5 mmol/L (193 mg/dL)							
Yes	2706	80.0	8971	80.6	11,677	80.4	<0.001
No	475	14.0	1826	16.4	2301	15.9	
Not reported	201	5.9	338	3.0	539	3.7	
Mean (SD) in mmol/L	3181	4.2 (0.94)	10,797	4.3 (1.57)	13,978	4.2 (1.45)	<0.001
Mean in mg/dL		162.4		166.3		162.4	
HbA1c (mmol/mol)							
≤59 (7.5%)	2179	64.4	6995	62.8	9174	63.2	0.362
59–≤64 (8.0%)	310	9.2	1054	9.5	1364	9.4	
65–≤75 (9.0%)	322	9.5	1074	9.6	1396	9.6	
>75	185	5.5	702	6.3	887	6.1	
Not reported	386	11.4	1310	11.8	1696	11.7	
Mean (SD)	2996	54.0 (12.5)	9825	54.5 (13.1)	12,821	54.3 (13.0)	0.060
Mean (%)		7.1		7.1		7.1	

*SD* standard deviation, *HbA1c* glycosylated hemoglobin

The total number of years of follow-up in the combined cohort was 59,431, 13,372 (22.5%) of which were for cancer patients. Overall, the median length of follow-up was 1495 days (4.1 years), and follow-up was statistically significantly shorter in cancer patients (median 1444 days) than in controls (median 1511 days: log-rank test for equality of survivor functions, *p* < 0.0001).

### Diabetes complications

**Fig. 1:**
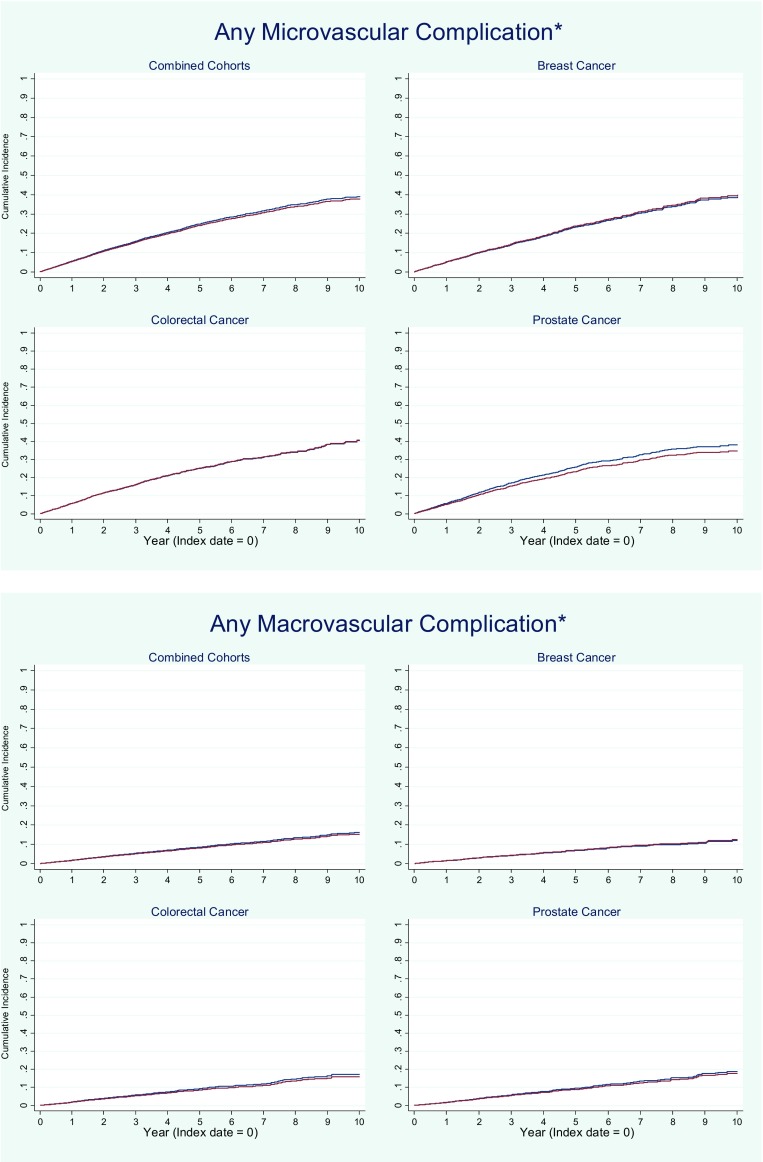
Cumulative incidence of microvascular and macrovascular complications. *Asterisk*, microvascular complications consisted of retinopathy, neuropathy, nephropathy, or chronic kidney disease (stage 4 or 5). Macrovascular complications consisted of peripheral arterial disease, acute myocardial infarction or coronary syndrome, cerebrovascular accident, or lower limb amputation. Cancer patients (*red*) and controls (*blue*)

There were no differences between cancer patients and controls in the adjusted rate of any microvascular or macrovascular complication (Fig. 1), either in the combined cohort or in any of the three cancer cohorts (total of 80 unadjusted and adjusted comparisons), except that in the combined cohort and only in the colorectal cancer cohort, cancer patients were less likely to develop retinopathy (adjusted HRs 0.88; 95% CI = 0.79–0.98 and 0.81; 95% CI = 0.66–0.91, respectively). The results of sensitivity analyses in which a variable specifying the type of diabetes (type I or type II) was added to the list of predictors in the adjusted models were virtually identical to those reported above (results not shown) (Table 2).

**Table 2:** Multivariate analyses of complications

Type of complication	Cohort							
	Combined		Breast		Colorectal		Prostate	
	Unadjusted hazard ratio [95% CI]	Adjusted hazard ratio [95% CI]	Unadjusted hazard ratio [95% CI]	Adjusted hazard ratio [95% CI]	Unadjusted hazard ratio [95% CI]	Adjusted hazard ratio [95% CI]	Unadjusted hazard ratio [95% CI]	Adjusted hazard ratio [95% CI]
Any microvascular	0.96 [0.87–1.06]	0.99 [0.89–1.09]	1.03 [0.86–1.23]	1.04 [0.86–1.25]	1.01 [0.85–1.20]	1.02 [0.85–1.22]	0.89 [0.76–1.05]	0.94 [0.80–1.11]
Chronic kidney disease	1.08 [0.85–1.36]	1.13 [0.89–1.43]	1.23 [0.83–1.82]	1.22 [0.81–1.82]	0.94 [0.62–1.44]	1.05 [0.68–1.64]	1.01 [0.68–1.51]	1.11 [0.74–1.68]
Nephropathy	1.12 [0.66–1.88]	1.19 [0.70–2.02]	1.16 [0.41–3.28]	1.19 [0.41–3.45]	1.71 [0.78–3.73]	1.82 [0.83–3.98]	0.65 [0.25–1.70]	
Neuropathy	1.07 [0.88–1.31]	1.10 [0.90–1.35]	1.22 [0.85–1.76]	1.23 [0.85–1.78]	1.31 [0.96–1.81]	1.30 [0.94–1.80]	0.83 [0.59–1.17]	0.84 [0.59–1.18]
Retinopathy	0.86** [0.77–0.96]	0.88* [0.79–0.98]	0.96 [0.79–1.17]	0.97 [0.79–1.18]	0.82 [0.67–1.00]	0.81* [0.66–0.99]	0.83* [0.69–0.99]	0.89 [0.74–1.06]
Any macrovascular	0.94 [0.80–1.11]	0.96 [0.82–1.13]	1.04 [0.77–1.40]	1.00 [0.74–1.37]	0.92 [0.69–1.21]	0.94 [0.71–1.24]	0.93 [0.72–1.21]	0.98 [0.75–1.26]
Acute myocardial infarction	1.09 [0.87–1.37]	1.11 [0.88–1.39]	0.82 [0.50–1.32]	0.79 [0.48–1.29]	1.42 [0.99–2.04]	1.37 [0.94–1.99]	1.03 [0.72–1.49]	1.12 [0.77–1.62]
Cerebrovascular accident	0.99 [0.78–1.25]	0.99 [0.78–1.26]	1.33 [0.87–2.03]	1.42 [0.92–2.17]	0.90 [0.59–1.37]	0.97 [0.64–1.47]	0.87 [0.59–1.28]	
Lower limb amputation	0.76 [0.45–1.29]	0.83 [0.49–1.42]	0.51 [0.11–2.28]	0.45 [0.10–2.09]	1.18 [0.56–2.49]	1.21 [0.53–2.74]	0.55 [0.23–1.31]	0.62 [0.25–1.52]
Peripheral arterial disease	0.74* [0.56–0.97]	0.78 [0.59–1.03]	0.99 [0.56–1.74]	0.94 [0.52–1.72]	0.60* [0.36–1.00]	0.66 [0.39–1.10]	0.79 [0.52–1.18]	0.86 [0.57–1.30]

*CI* confidence interval

*p < 0.05; **p < 0.01

#### Mortality

All-cause mortality was statistically significantly higher in cancer patients than controls (Table 3). However, there was no evidence cancer adversely impacted diabetes-related mortality. Diabetes-related mortality was significantly lower among cancer patients in the combined cohort (adjusted HR 0.76; 95% CI = 0.61–0.94) and in the prostate cancer cohort (adjusted HR 0.61; 95% CI = 0.43–0.88) (Table 3). The results of sensitivity analyses in which a variable specifying the type of diabetes (type I or type II) was added to the list of predictors in the models were virtually identical to those reported above (results not shown).

**Table 3:** Multivariate survival analyses

	Cohort							
	Combined		Breast		Colorectal		Prostate	
	Unadjusted hazard ratio [95% CI]	Adjusted hazard ratio [95% CI]	Unadjusted hazard ratio [95% CI]	Adjusted hazard ratio [95% CI]	Unadjusted hazard ratio [95% CI]	Adjusted hazard ratio [95% CI]	Unadjusted hazard ratio [95% CI]	Adjusted hazard ratio [95% CI]
Mortality								
All cause	1.47*** [1.33–1.63]	1.57*** [1.41–1.74]	1.47** [1.21–1.80]	1.52*** [1.24–1.85]	1.64*** [1.38–1.95]	1.71*** [1.43–2.04]	1.44*** [1.22–1.72]	1.60*** [1.35–1.91]
Diabetes	0.73** [0.59–0.90]	0.76*** [0.61–0.94]	0.87 [0.58–1.31]	0.91 [0.59–1.40]	0.88 [0.63–1.23]	0.87 [0.62–1.23]	0.57** [0.40–0.82]	0.61*** [0.43–0.88]

**p < 0.01; ***p < 0.001

## Discussion

Evidence indicates that preexisting diabetes is associated with worse outcomes in several types of cancer. However, there is less information on whether incident cancer is associated with worse diabetes outcomes. This as an important gap because as the relative survival for many types of cancer continues to improve, overall survival depends increasingly on the quality and outcomes of care for other underlying conditions. Overall, this study suggests that in the UK, there is no reason to suspect that the outcomes of diabetes in the presence of cancer are adversely affected by the competing needs of cancer care.

Among 80 unadjusted and adjusted comparisons of diabetes complications, we found no instance in which cancer was associated with higher incidence of a complication. Cancer was associated with lower adjusted incidence of retinopathy in the colorectal cancer and the combined cohorts, but the reason for this is not immediately clear, and chance finding due to multiple testing cannot be ruled out. As might be expected, cancer was associated with higher all-cause mortality even though we excluded patients who survived less than 1 year. However, there was no evidence of an adverse impact on diabetes-related mortality. In fact, our findings suggest that prostate cancer was associated with lower diabetes mortality in competing risks regression that accounted for death due to other causes.

There are several possible reasons that we found no adverse associations between cancer and diabetes complications or mortality. First, we were able to adjust for differences in baseline characteristics between diabetic cancer patients and diabetic noncancer controls that otherwise could have confounded associations between cancer and diabetes outcomes. These included baseline BMI, smoking status, HbA1c, cholesterol, and blood pressure. Second, our study was conducted in the UK, which has a robust primary care system in which, under the quality and outcomes framework [33], there are financial incentives for GPs to provide high-quality diabetes care throughout the continuum of care for other conditions such as cancer.

Our study has several strengths, which are attributable largely to the high quality of the database we used. Notably, because the study was based on CPRD, we were able to adjust for additional clinical factors typically not available in US health insurance claims databases, which otherwise may have confounded associations between cancer and the outcomes of diabetes. These included baseline BMI, smoking status, blood pressure, cholesterol, and HbA1c. In addition, we had rich data about clinical outcomes over a relatively long period of time. Our study also has several limitations. First, at the time it was conducted, we were unable to link the CPRD data to information from the National Cancer Intelligence Network (NCIN), which would have given us details on cancer stage and initial treatment. Although primary care data have a high sensitivity and specificity for identifying cancer [42], registry data would have allowed us to exclude cancer patients diagnosed with metastatic disease. We considered using Read codes in the primary care data files or ICD-10 codes in the Hospital Episode Statistics (HES) inpatient data to stage patients. However, we are not aware of any studies in the UK that validate the use of ICD-10 codes for this purpose, and because only two thirds of the patients in our study were linked to HES, doing so would have limited our sample sizes for all the analyses. Instead, we excluded patients who died within the first year after their index date. Second, since we did not have access to cancer treatment data, we were unable to identify subgroups of cancer patients who may have been at higher risk of diabetes complications or related mortality due to the cancer treatment they received. This should be the subject of further research once linkage to NCIN and chemotherapy databases becomes more readily available. Third, although we required all patients to have survived at least 1 year after their index date, requiring longer survival might have enabled us to better assess the longer-term effects of cancer treatment among the cancer patients. Finally, our findings may not be generalizable to long-term survivors of breast, colorectal, and prostate cancer in an era of full implementation of QOF, which was designed to improve the quality of diabetes primary care, to other countries that do not have primary care performance measures for diabetes care in place, or to other types of cancers.

## Conclusions

Overall, incident cancer appears to have had little adverse impact on the long-term outcomes of preexisting diabetes during 10 years after a diagnosis of breast, colorectal, or prostate cancer. These findings are important for cancer survivors with preexisting diabetes because they suggest that the advances in cancer therapy and supportive care, which have substantially improved the relative survival of several of the most common types of cancer, are not undermined by excess diabetes morbidity and mortality.
